# Purification and Biochemical Characterisation of Rabbit Calicivirus RNA-Dependent RNA Polymerases and Identification of Non-Nucleoside Inhibitors

**DOI:** 10.3390/v8040100

**Published:** 2016-04-14

**Authors:** Nadya Urakova, Natalie Netzler, Andrew G. Kelly, Michael Frese, Peter A. White, Tanja Strive

**Affiliations:** 1 Commonwealth Scinetific and Industrial Research Organisation, Health and Biosecurity, 2601 Canberra, ACT, Australia; nadezda.urakova@csiro.au (N.U.); michael.frese@canberra.edu.au (M.F.); 2 Invasive Animals Cooperative Research Centre, University of Canberra, 2617 Canberra, ACT, Australia; 3 Health Research Institute, University of Canberra, 2617 Canberra, ACT, Australia; 4 School of Biotechnology and Biomolecular Sciences, Faculty of Science, University of New South Wales, 2052 Sydney, NSW, Australia; n.netzler@unsw.edu.au (N.N.); agk22000@gmail.com (A.G.K.); p.white@unsw.edu.au (P.A.W.); 5 Institute for Applied Ecology, University of Canberra, 2617 Canberra, ACT, Australia

**Keywords:** RHDV, RCV-A1, polymerase, non-nucleoside inhibitors, antiviral agents

## Abstract

Rabbit haemorrhagic disease virus (RHDV) is a calicivirus that causes acute infections in both domestic and wild European rabbits (*Oryctolagus cuniculus*). The virus causes significant economic losses in rabbit farming and reduces wild rabbit populations. The recent emergence of RHDV variants capable of overcoming immunity to other strains emphasises the need to develop universally effective antivirals to enable quick responses during outbreaks until new vaccines become available. The RNA-dependent RNA polymerase (RdRp) is a primary target for the development of such antiviral drugs. In this study, we used cell-free *in vitro* assays to examine the biochemical characteristics of two rabbit calicivirus RdRps and the effects of several antivirals that were previously identified as human norovirus RdRp inhibitors. The non-nucleoside inhibitor NIC02 was identified as a potential scaffold for further drug development against rabbit caliciviruses. Our experiments revealed an unusually high temperature optimum (between 40 and 45 °C) for RdRps derived from both a pathogenic and a non-pathogenic rabbit calicivirus, possibly demonstrating an adaptation to a host with a physiological body temperature of more than 38 °C. Interestingly, the *in vitro* polymerase activity of the non-pathogenic calicivirus RdRp was at least two times higher than that of the RdRp of the highly virulent RHDV.

## 1. Introduction

Rabbit haemorrhagic disease (RHD) is a highly infectious disease with high case fatality and morbidity rates in adult rabbits. The incubation period ranges between 1 and 3 days and rabbits usually die within 12–36 h after the onset of fever, which is not always observed [1,2,3]. The primary target organs for RHD are the liver, lungs and the spleen, with involvement of the resident macrophages [4]. The major histopathological features of RHD include acute hepatitis and splenomegaly. Haemorrhages and congestions can be found in a variety of organs, particularly in the lungs, heart and kidneys, as a result of massive disseminated intravascular coagulation [1,5]. Rabbit haemorrhagic disease virus (RHDV) infection in rabbits causes severe leukopenia, involving B and T lymphocytes [6,7], and granulocytes [6]. Depletion of peripheral blood leucocytes and lymphocytes in the liver and spleen accompanies the disease and is associated with apoptosis [7,8].

RHD was first detected in China in 1984 [3] and subsequently disease outbreaks were reported from most continents and in many countries, including Korea, Italy, Spain, Portugal, France, United Kingdom, Mexico, North America, Cuba, several countries in Africa and elsewhere (reviewed in [2]). In the Iberian Peninsula, where European rabbits originated and where they constitute a keystone species of the ecosystem, RHD caused and continues to cause severe reductions of wild rabbit populations. This in turn has affected many predator species, e.g. the critically endangered Spanish imperial eagle and the Iberian lynx [9,10]. The real impact of RHD on local ecosystems can be much greater, since rabbits alter plant species composition and vegetation structure through grazing and seed dispersal [9]. In Australia and New Zealand, RHDV has been utilized since the mid-1990s as a biological control agent for rabbits, a serious pest in these countries, resulting in substantial benefits to agricultural industries and the environment [11,12].

RHDV is a single-stranded positive-sense RNA virus [2,13,14] that belongs to the family *Caliciviridae*, genus *Lagovirus* [15,16]. Its RNA is tightly packaged into non-enveloped icosahedral capsids that consist of 180 VP60 proteins [2,17]. The 35-nm virions contain 7.4 kb of genomic RNA and additional 2.1 kb fragments of subgenomic RNA that are collinear with the 3′ end of the genomic RNA [14,18,19]. Both genomic and subgenomic viral RNAs are polyadenylated at the 3′ end [2] and covalently linked to the genome binding protein (VPg) at the 5′ end [2,19,20]. The genomic RNA contains two slightly overlapping reading frames (ORF) of 7 kb (ORF1) and 351 nucleotides (ORF2) [13,14]. ORF1 is translated into a large polyprotein that is cleaved into several non-structural proteins and the major structural protein, the capsid protein [13,18,21]. ORF2 encodes a minor structural protein, VP10 [2,13]. The subgenomic RNA only encodes both the structural proteins, VP60 [2,22] and VP10 [2]. The RHDV genome encodes a total of at least nine proteins [13,18]; comprising the helicase, the VPg protein, the protease, the RNA-dependent RNA polymerase (RdRp), the capsid protein VP60, the VP10 protein and three proteins of unknown function, p16, p23 and p29 [2,13,18,23].

**Figure 1:**
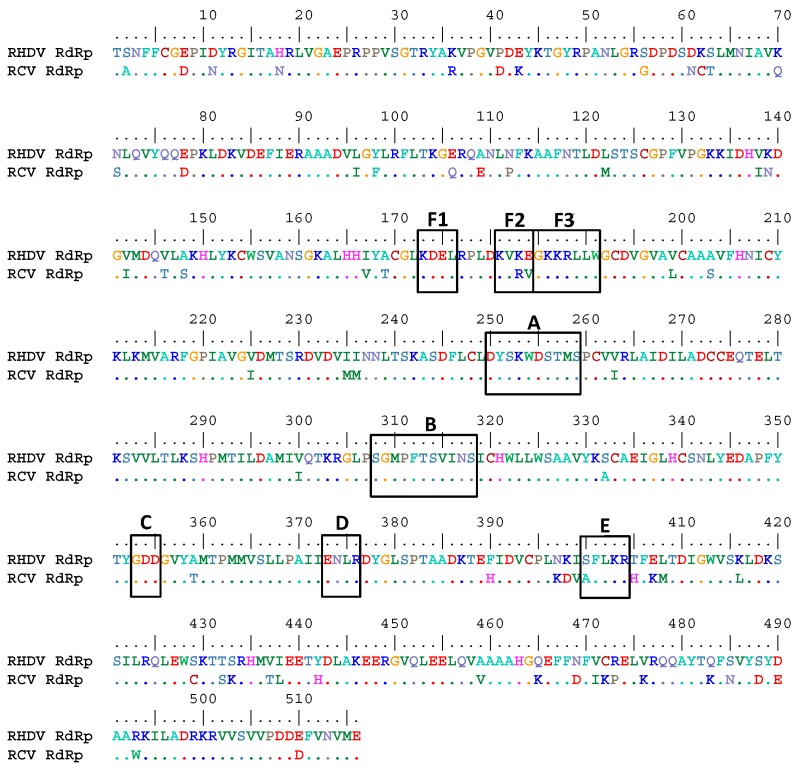
Amino acid alignment of RHDV and RCV RdRps. The alignment compares RHDV Czech strain V351 (GenBank accession number KF594473.1) and RCV-A1 (GenBank accession number EU871528.1) and was conducted with the BioEdit software. Conserved motifs (**A**–**E,** **F1**–**F3**) attributed to RdRps of single-stranded positive-sense RNA viruses [30] are shown in black boxes.

The emergence of RHDV from a pre-existing non-pathogenic rabbit calicivirus that became a lethal pathogen by mutation and subsequently spread around the world, has been suggested [24]. Indeed benign rabbit caliciviruses (RCVs), which are non-pathogenic relatives of RHDV, have been discovered in Australia [25] and several European countries [26,27]. In contrast to RHDV, RCV strains do not target the liver or cause disease in rabbits, but lead to a localised, subclinical infection of the small intestine [28,29]. Despite these significant differences in the viral pathology, the genome organisation and the amino acid sequences of pathogenic and non-pathogenic viruses are very similar; e.g. the RHDV and RCV RdRps, key enzymes in the replication cycle of rabbit caliciviruses, show up to 90% amino acid identity (Figure 1).

In 2010, a new RHDV variant (RHDV2) that caused atypical RHD outbreaks among vaccinated [31] and young [32,33] rabbits emerged in France [31]. This is remarkable because both are usually refractory to lethal RHDV infection. The subsequent analysis of a series of RHDV2 full length genomes revealed multiple recombination events that share a common theme, *i.e.*, all recombinants possessed the structural proteins of RHDV2 and the non-structural proteins of either a non-pathogenic rabbit calicivirus strain or from a pathogenic genogroup 1 strain [34]. Since existing vaccines are based on “classic” strains, RHDV2 quickly disseminated throughout Europe, killing a high proportion of vaccinated animals in affected rabbitries [35]. The existing vaccines appeared at least partially effective against RHDV2 [32,36]. However, there was no guaranteed way of reliably protecting valuable breeding stock and pets in the years between the detection of this new emerging rabbit calicivirus variant and the development of a specific vaccine, which has only recently become available [37]. RHDV2 has now spread beyond Europe and has recently been reported in Australia [38], where the new RHDV2 vaccine is not available yet and farmed and pet rabbits are currently at risk from this new emerging RHDV variant. Antivirals that could effectively treat infected rabbits would represent a useful additional tool to control outbreaks in high value rabbit breeding enterprises, and would also help to treat valued family pets until new vaccines become available.

The lack of an effective cell culture system for rabbit caliciviruses [2,39] prompted us to adopt cell-free *in vitro* assays for testing inhibitors of the RdRp, a protein that represents a prime target for antiviral drug design due to its essential role in the virus replication cycle and the fact that eukaryotic cells do not possess closely related enzymes. Sequence similarities between the 3D RdRp of picornaviruses and the RHDV polyprotein cleavage product p58 suggest that both polypeptides have a similar role in genome replication [40,41]. Expression of the respective coding region in *Escherichia coli (E. coli)* showed that p58 is indeed an enzymatically active RdRp [40], and did not demonstrate DNA-dependent RNA polymerase, reverse transcriptase or DNA-dependent DNA polymerase activities [41]. Crystal structure of RHDV RdRp revealed that this enzyme adopts a shape that resembles a right hand, with domains corresponding to the fingers, palm and thumb, as seen in the three-dimensional structures of many other polymerases [42]. *In vitro* activity assays demonstrated that recombinant RHDV RdRp was able to use (+) and (–) single-stranded RNA templates in the absence of added primers and could synthesize subgenomic RNA by internal initiation of replication, using a subgenomic promoter on a (–) strand genomic RNA template [43]. It has also been reported that, in addition to its polymerase activity, p58 is able to catalyse VPg uridylylation [20].

In this study, recombinant RdRps from a pathogenic and a non-pathogenic rabbit caliciviruses were tagged with a C-terminal hexahistidine, expressed in *E. coli* and purified by nickel affinity chromatography. Basic enzyme characteristics (effects of temperature, MnCl_2_ concentration, pH and substrate concentrations) and the inhibitory effects of several non-nucleoside inhibitors (NNIs) were studied. These NNIs were previously identified through high-throughput screening as human norovirus (NoV) RdRp inhibitors [44].

Taking into account the recombination events that led to the evolution of RHDV2 [34], RdRps from both pathogenic and non-pathogenic strains were included in the study to identify compound(s) that can suppress both viral enzymes and thus can be potentially effective against any new RHDV variants.

## 2. Materials and Methods

### 2.1. Plasmids

RHDV RNA was purified from a commercial RHDV suspension (Czech strain V351, GenBank accession number KF594473.1, Elizabeth Macarthur Agricultural Institute, Menangle, Australia); RCV RNA was purified from the homogenised small intestine of a rabbit infected with the non-pathogenic calicivirus RCV-A1 (GenBank accession number EU871528.1) [28] using the RNeasy Mini Kit (Qiagen, Hilden, Germany). Viral RNA was converted into cDNA using SuperScript III reverse transcriptase (Life Technologies, Carlsbad, CA, USA). The cDNAs encoding viral RdRps were amplified using Q5 High-Fidelity DNA polymerase (New England BioLabs, Ipswich, MA, USA) and gene-specific primers (GeneWorks; for sequence information see Table 1), according to the instructions of the manufacturer. Resulting amplicons were cloned into the pET-26b(+) expression vector (Novagen, Darmstadt, Germany) to generate recombinant polymerases with a C-terminal His_6_-tag using the following strategy. PCR products were digested using the *Xho*I restriction enzyme (New England BioLabs). The pET-26b(+) expression vector was linearised using the *Nde*I restriction enzyme (New England BioLabs), 5′ overhangs were filled in using T4 DNA polymerase (Promega, Madison, WI, USA) to generate a blunt end and was further digested using the *Xho*I restriction enzyme. All inserts were ligated into linearised vectors using the T4 DNA ligase (New England BioLabs).

NoV GII.4 Sydney 2012 RNA (NoV NSW028D/January/2013, GenBank accession number KT239579) was purified from a clinical stool sample, converted into cDNA, amplified and cloned into the pGEM-T-easy vector. The full length coding sequence of NoV GII.4 RdRp was amplified from the resulting plasmid using gene-specific primers (Table 1) and subsequently cloned into pET-26b(+) expression vector using *Nde*I (5′ terminus) and *Xho*I (3′ terminus) restriction sites.

### 2.2. Compounds

**Figure 2:**
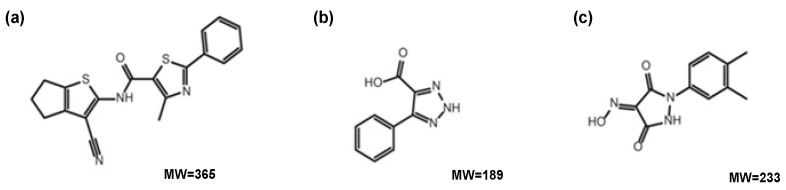
Chemical structure of compounds tested as potential antiviral agents against rabbit caliciviruses (from [44]). (**a**) NIC02; (**b**) NIC10; (**c**) NIC12. *M*w—molecular weight, g/mol.

Compounds that were tested in the study: NIC02 (a phenylthiazole-5-carboxamide), NIC10 (a triazole), NIC12 (a pyrazolidine-3,5-dione) (Figure 2) and NIC12 derivatives (NIC12-2, NIC12-3, NIC12-4, NIC12-5) were described previously [44].

### 2.3. Protein Expression and Purification

The constructs encoding His_6_-tagged RdRps were transformed into *E. coli* BL21(DE3) cells (BioLabs). Selected colonies were suspended in 10 mL of Luria-Bertani (LB) medium with 50 μg/mL kanamycin (Sigma-Aldrich, St. Louis, MO, USA) and incubated at 37 °C, in an orbital shaker at 200 *r.p.m*. for 6 h. Prepared cultures (10 mL) were inoculated into 500 mL of LB-kanamycin medium and incubated at 37 °C, 200 *r.p.m*., until the OD_600_ reached 0.5. Expression of the RdRps was induced with 1 mM isopropyl-β-d-thiogalactopyranoside (IPTG) (Sigma-Aldrich) and the culture was further incubated at 25 °C overnight. Cells were collected by centrifugation and the bacterial pellet was stored at −80 °C until use.

For RdRp purification, bacterial pellets were resuspended in 1× CelLytic™ B buffer (Sigma-Aldrich) containing 25 mM Tris-HCl (pH 8), 50 mM NaCl (for RHDV/RCV RdRps) or 10 mM NaCl (for NoV RdRp), 100 mg/mL of lysozyme (Sigma-Aldrich), 50 U/mL of Benzonase^®^ (Sigma-Aldrich), 2 mM MgCl_2_, 4 μg/mL RNase A (Sigma-Aldrich), and 1× ethylenediaminetetraacetic acid (EDTA)-free protease inhibitor cocktail solution (Sigma-Aldrich). Resuspended cells were incubated at room temperature for 30 min with shaking to allow for chemical lysis. After lysis, NaCl concentration was adjusted to 300 mM NaCl (RHDV/RCV RdRps) and lysates were clarified by centrifugation at 4 °C. The clarified lysate was loaded onto Ni^2+^ columns pre-equilibrated with loading buffer containing 25 mM Tris-HCl (pH 8), 300 mM (RHDV/RCV RdRps) or 500 mM (NoV RdRp) NaCl, and 0.2% octyl-P-glucoside (Sigma-Aldrich). Once bound, the columns were washed with loading buffer and then wash buffer containing 25 mM Tris-HCl (pH 8), 300 mM (RHDV/RCV RdRps) or 500 mM (NoV RdRp) NaCl, 0.1% octyl-P-glucoside and 10 mM (RHDV/RCV RdRps) or 5 mM (NoV RdRp) of imidazole. The RdRps were then eluted by increasing the imidazole concentration from 10 mM (RHDV/RCV RdRp) or 5 mM (NoV RdRp) to 300 mM (RHDV/RCV RdRp) or 500 mM (NoV RdRp). The purified RdRps were dialysed and stored in a buffer containing 150 mM NaCl, 50 mM Tris-HCl (pH 8.0), 10 mM MgCl_2_ (RHDV/RCV RdRps) or 150 mM NaCl, 25 mM Tris-HCl (pH 7.5), 1 mM EDTA, 1 mM dithiothreitol (DTT) (NoV RdRp) and 20% glycerol at −80 °C. The purity of RdRps was confirmed by SDS-PAGE. Protein concentration was estimated with a Nanodrop ND-1000 spectrophotometer (the protein A_280_ method).

### 2.4. Kinetics of RdRp Activity

Main RdRp activity was quantified by monitoring the formation of double-stranded RNA (dsRNA) from a single-stranded homopolymeric template, poly(C) (Sigma-Aldrich), using the fluorescent dye PicoGreen (Life Technologies), as described previously [45] with minor modifications. Briefly, RdRp activity assays were performed in 25-μL reactions. A standard reaction contained 20 ng/μL poly(C) RNA (Sigma-Aldrich), 0.5 mM rGTP (Promega), 2.5 mM MnCl_2_, and 5 mM DTT in 20 mM Tris-HCl (pH 7.5) buffer. Reactions were initiated by the addition of 20–40 ng RdRp and incubated at 30 °C for 15 min unless stated otherwise. Reactions were terminated with 5 mM EDTA, followed by PicoGreen staining and dsRNA quantitation using either POLARstar or SpectraMax M3 plate readers at standard wavelengths (excitation 480 nm, emission 520 nm). Control reactions stopped at 0 min were used to quantify background fluorescence.

The optimum conditions for RdRp activities were examined by titrating enzyme concentrations, incubation time, concentration of MnCl_2_ (0–10 mM), varying pH (7.0–9.5, 20 mM Tris-HCl) and reaction temperatures (4–60 °C). To evaluate the kinetics of nucleotide incorporation by the RCV and RHDV RdRps, reactions were run with 40 ng of enzyme, 2.5 mM MnCl_2_, 5 mM DTT and 20 ng/μL poly(C) in 20 mM Tris-HCl (pH 7.5) buffer for 15 min at 30 °C with increasing rGTP concentrations (0–2.0 mM). To evaluate the kinetics of the poly(C) template utilisation, reactions were run with 40 ng of enzyme, 2.5 mM MnCl_2_, 5 mM DTT and 0.5 mM rGTP substrate in 20 mM Tris-HCl buffer (pH 7.5) for 15 min at 30 °C with increasing poly(C) concentrations (0–80 ng/μL).

### 2.5. Quantitative RdRp Assays to Test Non-Nucleoside Inhibitors (NNI)

RdRp assays were performed in 384-well plates, as described previously [45]. A single 25-μL reaction mixture contained 20–400 ng enzyme, 45 μM rGTP, 10 ng/μL poly(C) RNA, 2.5 mM MnCl_2_, 5 mM DTT, 0.01% bovine serum albumin (BSA), and 0.005% Tween 20 in 20 mM Tris-HCl (pH 7.5). RdRps were incubated for 10 min at 30 °C in the presence of the test compounds or the compound vehicle DMSO (0.5% *v/v*) before addition into the reaction mixture. The reaction was then allowed to proceed for 15 min at 30 °C. The reaction was terminated with 5 mM EDTA, followed by PicoGreen staining and dsRNA quantitation using a POLARstar plate reader at standard wavelengths (excitation 480 nm, emission 520 nm).

### 2.6. Gel-Based RdRp Assays

Denaturing polyacrylamide gel-based assays were used to examine the primer extension activity of the RdRps, using a previously described method [46] with modifications. The original template PE46 was modified to replace 13 nucleotides at the 5′ end with 13 cytosines, resulting in a new template PE46-C. The new template PE46-C (5′-CCCCCCCCCCCCCCCAUAUACUUCGGUAUAUGG-3′) was used to direct primer extension activity through a stable hairpin at the 3′ end. Reactions were performed using 1 µM PE46-C template and 400 ng RdRp, 0.4 mM rGTP (Promega), 5 mM DTT, 2.5 mM MnCl_2_, and 20 mM Tris-HCl (pH 7.5) in a final volume of 25 µL. RdRps were incubated for 10 min at 30 °C in the presence of the test compounds or the compound vehicle DMSO (0.5% *v/v*) prior to addition into the reaction mixture. Reaction mixtures were then incubated for 6 h at 30 °C. RNA products were analysed on 15% denaturing polyacrylamide gels containing 7 M urea (Bio-Rad, Hercules, CA, USA). Gels were stained with SYBR green II (Invitrogen) and visualised on a Gel Doc molecular imager (Bio-Rad).

### 2.7. Data Analysis

Amino acid alignments were conducted with BioEdit software. Enzyme kinetics data were analysed and graphs were generated using Graphpad Prism software (version 6.05).

## 3. Results

### 3.1. RdRp Expression and Characterisation

To investigate the biochemical properties of rabbit calicivirus RdRps and to test potential inhibitors, the entire RdRp coding regions from a pathogenic RHDV strain (Czech V351) and that of a non-pathogenic RCV strain (RCV-A1) were cloned into a bacterial expression vector, which allowed the production of recombinant proteins with a C-terminal leucine-glutamic acid followed by a hexahistidine tag to facilitate nickel affinity purification. The identity and quality of the purified proteins was confirmed by SDS-PAGE, Western blot and tandem mass spectrometry of tryptic peptides [47] (data not shown).

**Figure 3:**
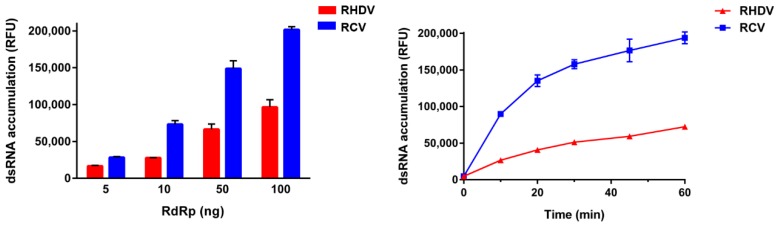
Comparison of RHDV and RCV RdRp activities. Purified recombinant RdRps were used to generate dsRNA from a poly(C) RNA (20 ng/μL) template using rGTP (0.5 mM) as substrate. Following incubation at 30 °C, reactions were stopped with 5 mM EDTA and dsRNA was quantified using the fluorescent dye PicoGreen. RHDV RdRp activity is shown in red, RCV RdRp activity is shown in blue. The results from a representative experiment are shown with average values and standard deviations from triplicate reactions for each measurement point. (**a**) The effect of RHDV and RCV RdRp concentrations on dsRNA formation over 15 min; (**b**) The synthesis of dsRNA catalysed by 20 ng of RHDV and RCV RdRps over a 1-h period. RFU—relative fluorescence units.

To characterise the kinetic properties of RHDV and RCV-A1 RdRps and to compare the performance of these polymerases with that of other virus polymerases (e.g., human NoV RdRp), polymerase activities were measured using a fluorescence-based *in vitro* assay under a variety of conditions, including different pH, temperature, MnCl_2_ concentration, template and substrate concentrations. Interestingly, the *in vitro* polymerase activity of the RCV enzyme was at least two times higher than that of the RHDV RdRp activity (Figure 3).

**Figure 4:**
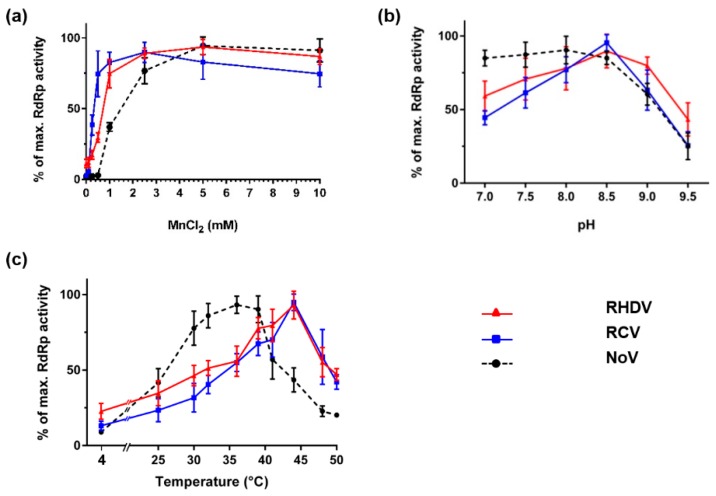
Impact of MnCl_2_, pH and temperature on calicivirus RdRp activity. Purified recombinant RdRps (40 ng of RHDV and RCV RdRps and 200 ng of NoV RdRp) were used to generate dsRNA with poly(C) RNA (20 ng/μL) as template and rGTP (0.5 mM) as substrate. Following a 15-min incubation in the presence of different (**a**) MnCl_2_ concentrations; (**b**) pH levels; or (**c**) temperatures, reactions were stopped with EDTA at a final concentration 5 mM and dsRNA was quantified using the PicoGreen reagent. Unless indicated otherwise, reactions were incubated at 30 °C. RHDV RdRp activity is shown in red as triangles, RCV RdRp activity is shown in blue as squares and NoV RdRp activity is shown in black as circles. Averages of relative fluorescence levels were calculated and plotted with standard deviations. The results were generated from two (**a**) or three (**b** and **c**) independent experiments with triplicate reactions for each measurement point.

To determine the optimal concentration of MnCl_2_ for the *de novo* RdRp activity on a poly(C) RNA template, RdRp assays with MnCl_2_ concentrations ranging from 0 to 10 mM were conducted. RdRp activities increased with increasing concentrations of MnCl_2_ in the range 0 to 2.5 mM and reached a plateau at higher concentrations (Figure 4a). RCV and RHDV RdRp activities were also tested with 20 mM Tris-HCl reaction buffers adjusted to pH 7.0, 7.5, 8.0, 8.5, 9.0 and 9.5. Both RHDV and RCV RdRps showed the highest activities at pH 8.5. Compared to NoV RdRp, that showed similar activities at pH ranging from 7.0 to 8.0, the pH optimum for RHDV and RCV RdRps activities were found to be highest at the relatively basic value of pH 8.5 (Figure 4b). Next we assessed RCV and RHDV RdRps for *de novo* polymerase activity at different temperatures ranging from 4 °C to 50 °C. Compared to NoV RdRp, that showed the highest activities between 35 and 39 °C, rabbit calicivirus RdRps were more active at higher temperatures. Both RdRps showed the highest activities between 40 and 45 °C. Strong activities, approximately 50% and more of the highest values, were exhibited by both enzymes at temperatures ranging from 30 to 50 °C (Figure 4c).

**Figure 5:**
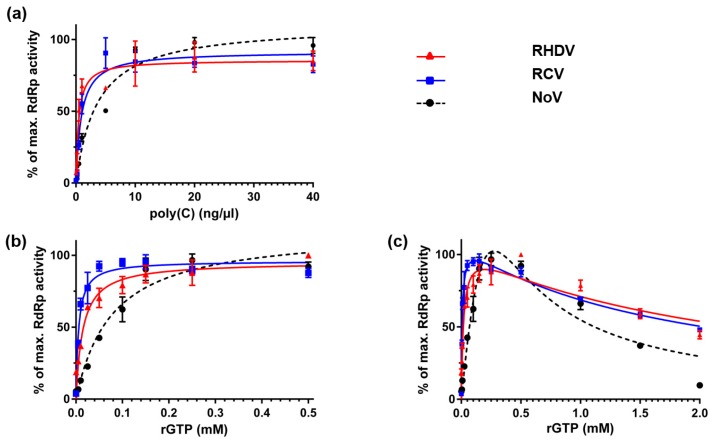
Kinetics of template and substrate utilisation by calicivirus RdRps. Purified recombinant RdRps (40 ng of RHDV and RCV RdRps and 200 ng of NoV RdRp) were used to generate dsRNA with poly(C) RNA as template and rGTP as substrate. (**a**) The kinetics of template utilisation was examined by titrating poly(C) from 0 to 40 ng/μL in the presence of 0.5 mM rGTP; (**b**,**c**) The kinetics of nucleotide incorporation was examined by titrating rGTP from 0 to 0.5 mM and 0 to 2.0 mM in the presence of 20 ng/μL of poly(C). Following a 15-min incubation at 30 °C, reactions were stopped with EDTA at final concentration 5 mM and dsRNA was quantified using the PicoGreen reagent. RHDV RdRp activity is shown in red as triangles, RCV RdRp activity is shown in blue as squares and NoV RdRp activity is shown in black as circles. The results from a representative experiment are shown with average values and standard deviations from triplicate reactions for each measurement point.

To evaluate the kinetics of template utilisation, reactions were run with increasing poly(C) concentrations (0–40 ng/μL). The results were in general agreement with the Michaelis-Menten model for enzyme kinetics. That is, RdRp activities went up with increasing concentrations of poly(C) in the range of 0 to 10 ng/μL and reached a plateau at higher concentrations (Figure 5a). To examine the kinetics of nucleotide incorporation by the RCV and RHDV RdRps, reactions were run with increasing rGTP concentrations (0–2.0 mM). Interestingly, in contrast to poly(C) template utilisation, the kinetics of rGTP polymerisation in the given range deviated from standard Michaelis-Menten kinetics. RdRp activities increased sharply in the range of 0 to 0.1 mM, plateaued between 0.1 and 0.5 mM and decreased at higher substrate concentrations (Figure 5b,c). Kinetic data for RHDV, RCV and NoV RdRps are shown in Table 2.

### 3.2. Testing of RdRp Inhibitors

**Figure 6:**
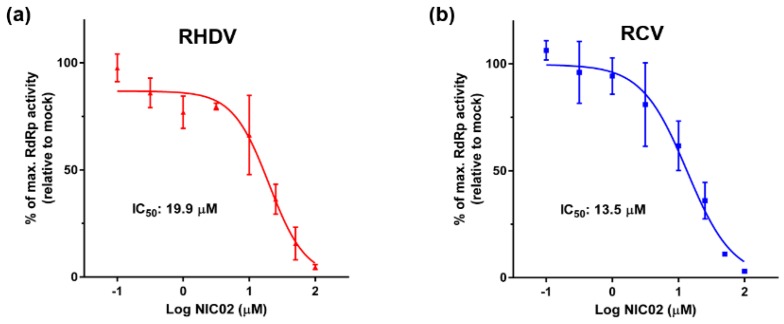
NIC02 inhibits *de novo* polymerase activity of rabbit calicivirus RdRps *in vitro*. The inhibitory effects of NIC02 on the *de novo* activity of (**a**) RHDV; and (**b**) RCV RdRps were analysed by monitoring the formation of dsRNA from a single-stranded poly(C) homopolymeric template. NIC02 concentrations were varied between 0.1–100 μM and compared to the relative activity in mock-treated samples containing the vehicle (0.5% *v/v* DMSO) only. Log (inhibitor concentration) *vs.* response curves were plotted. RHDV RdRp activity is shown in red as triangles, RCV RdRp activity is shown in blue as squares. Averages of relative fluorescence levels were calculated and plotted with standard deviations. Results from three independent experiments with triplicate reactions for each measurement point are shown.

**Figure 7:**
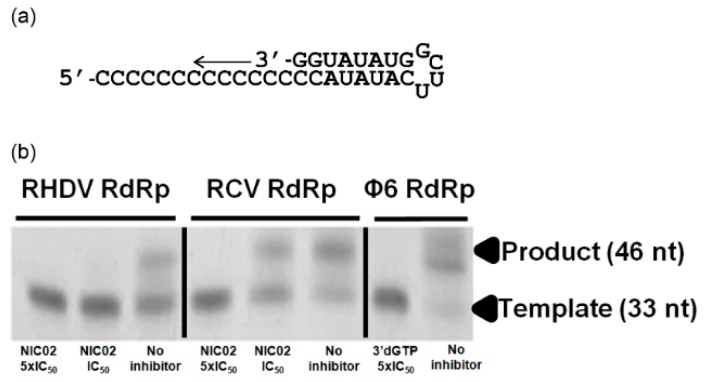
NIC02 inhibits primed elongation activity of rabbit calicivirus RdRps *in vitro*. A gel-based RdRp activity assay was used to evaluate effects of NIC02 on the primed elongation activity of RHDV and RCV RdRps. (**a**) PE46-C template. (**b**) Products generated in the in the presence of 19.9 (IC_50_) and 99.5 µM (5× IC_50_) NIC02 for RHDV RdRp, 13.5 (IC_50_) and 67.5 µM (5× IC_50_) NIC02 for RCV RdRp or in the presence of the vehicle (0.5% *v/v* DMSO) alone (no inhibitor). Products were separated on a 15% denaturing polyacrylamide gel and stained with SYBR green II. Inhibition of the RdRp of Φ6 bacteriophage was carried out using 200 µM of the nucleoside analogue chain terminator 3′dGTP as an inhibition control.

Three NNIs, namely NIC02, NIC10, and NIC12 were previously shown to inhibit the RdRp of NoV GII.4 (Den Haag 2006b variant) with the half-maximal inhibitory concentrations (IC_50_) of 5.0, 9.2 and 9.8 μM, respectively [44]. This prompted us to test these compounds against RCV and RHDV RdRps. NIC02 inhibited the *de novo* polymerase activity of both RHDV and RCV enzymes with the IC_50_ of 19.9 μM (95% CI: 9.1–57.0 μM) and 13.5 μM (95% CI: 10.9–29.0 μM), respectively (Figure 6). In addition, we found that NIC02 also inhibited the primed elongation activity of RHDV and RCV RdRps in a gel-based assay (Figure 7). Inhibition of RHDV RdRp by NIC02 is shown at 19.9 µM (IC_50_) and 99.5 µM (5× IC_50_) and RCV RdRp by NIC02 at 13.5 μM (IC_50_) and 67.5 µM (5× IC_50_). An attenuation of dsRNA formation is observed at the IC_50_ for both RHDV and RCV RdRps, with no dsRNA product visible at 5× the IC_50_ of NIC02.

**Figure 8:**
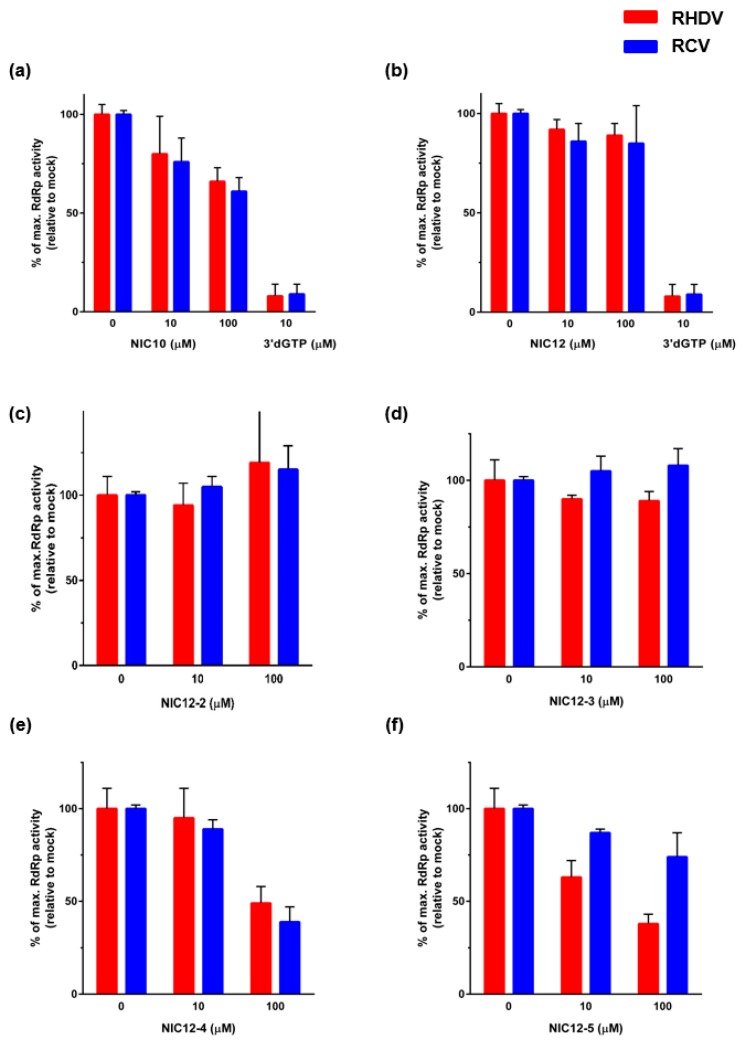
Limited inhibitory effects of NIC10, NIC12 and NIC12 derivatives on *de novo* polymerase activity of rabbit calicivirus RdRps *in vitro*. The effects of (**a**) NIC10; (**b**) NIC12 and the NIC12 derivatives; (**c**) NIC12-2; (**d**) NIC12-3; (**e**) NIC12-4; and (**f**) NIC-12-5 on the *de novo* activity of RHDV and RCV RdRps were analysed by monitoring the formation of dsRNA. The chain terminator 3′dGTP (10 µM) was used as a positive control (**a**,**b**). RHDV RdRp activity is shown in red, RCV RdRp activity is shown in blue. The results are averages of relative fluorescence levels determined from three independent experiments with triplicate reactions for each measurement point plotted with standard deviations.

In contrast, NIC10 and NIC12, as well as four structural derivatives of NIC12 (NIC12-2, NIC12-3, NIC12-4 and NIC12-5) showed only limited inhibitory effects on *de novo* RCV and RHDV RdRp activity (Figure 8).

## 4. Discussion

### 4.1. RdRp Expression and Characterisation

One of the hallmarks of RHDV is its extreme virulence and the fast course of the acute fatal disease (reviewed in [2]). Recent studies suggest that the virus is maintaining a high level of virulence and replication speed that enables the virus to kill susceptible hosts within 2–3 days, before adaptive immune responses develop [48]. In contrast, no pathogenicity has ever been observed in RCV-infected rabbits, although studies describing RCV infections show that localised replication in the small intestine also progresses quickly and virus shedding can be observed from day 1 post infection [28]. It was interesting to find that the *in vitro* polymerase activity of the non-virulent RCV RdRp was at least two times higher compared to the RdRp of the highly virulent RHDV, although other enzyme characteristics were very similar. An amino acid alignment of RHDV and RCV RdRps showed that they share up to 90% amino acid identity (Figure 1). However, it is notable that there are some amino acid substitutions in motifs E and F that appear to be conserved among RdRps from other single-stranded positive-sense RNA viruses [30,49,50]. It is a subject for further studies whether the observed differences in the activity of RHDV and RCV RdRps can be explained by the presence of amino acid substitutions in the E and F motifs (Figure 1). It is also presently unclear whether this interesting finding can be attributed to the physiological conditions under which rabbit caliciviruses replicate *in vivo*. It is tempting to speculate that the very high enzymatic activity of RCV RdRp is responsible for the high RCV genome load in the duodenum of infected rabbits (up to 9.4 × 10^6^ RNA copies per mg of tissue) despite the fact that only very few RCV antigen-positive epithelial cells can be detected in this tissue [29].

Previous studies have shown that RdRp fidelity can be a virulence determinant [51,52,53]. For the highly pathogenic RHDV, a reduced RdRp activity may therefore represent a mechanism to increase the fidelity of RNA synthesis, possibly assisting with maintaining very high levels of virulence in RHDV [48]. Trade-offs between replication speed and fidelity are important factors that shape virus evolution [54], and detailed comparative genetic studies of RCV-A1 and RHDV will be useful to assess any differences in the evolutionary rates of these viruses. These future studies would greatly benefit from *in vitro* studies that experimentally assess the fidelity of the two enzymes.

Our results revealed that salt requirements for *in vitro* activities of rabbit calicivirus RdRps are similar to those of human and murine calicivirus RdRps [55]. These similarities, however, do not extend to the pH requirements. In contrast to NoV RdRp that showed maximal activities between pH 7.0 and 8.0, rabbit calicivirus RdRps clearly prefer a more basic pH. This observation may be attributed to the properties of the tissues/cells in which RHDV and RCV replicate. Both viruses show a tropism for tissues involved in bicarbonate ion metabolism. The main replication site for RHDV is the liver, in particular the periportal areas, while RCV replicates in the duodenum. Therefore, local/intracellular pH in these tissues may be shifted towards a more basic pH [56,57,58].

A further unexpected result was that rabbit calicivirus RpRps are most active at temperatures between 40 and 45 °C. This observation is different from published data for many other RdRps that were found to work much more efficiently at lower temperatures (usually between 25 and 35 °C) [45,55,59,60,61,62,63]. However, it should be noted that, in our hands, NoV RdRp showed the highest activity between 35 and 39 °C, which is also higher compared to the previously published temperature optimum of 25 °C [55]. It should be further noted that our results were obtained using a fluorescence-based assay, whereas radioactive assays were used in earlier studies [55], a difference that may explain the observed discrepancies. It is generally accepted that viruses adapt to their hosts, so it should not be surprising if the optimal temperature for viral enzymes lie within the range of physiological temperatures observed in an infected host organism. Thus, the observed optimal *in vitro* temperatures for the NoV RdRp (35–39 °C) and rabbit calicivirus RdRps (40–45 °C) may reflect an adaptation to hosts with a body temperature of 37 °C (human) and more than 38 °C (rabbit), respectively. If our observation is indeed relevant to *in vivo* conditions*,* it can be speculated that the onset of fever (up to 42 °C [28]) during RHDV infection does not slow down virus replication.

The kinetics of the poly(C) template usage by both rabbit calicivirus RdRps tested in this study were in agreement with the Michaelis-Menten model for enzyme kinetics, while the kinetics of rGTP incorporation deviated from this model. Although in the range of substrate concentrations from 0 to 0.5 mM the results fit the Michaelis-Menten model, at higher substrate concentrations, a decrease in enzyme activity was observed. This phenomenon is referred to as substrate inhibition and is often interpreted as an abnormality that comes from using artificially high substrate concentrations in *in vitro* assays. However, examples that substrate inhibition can be a biologically relevant regulatory mechanism have been reported for a number of enzymes, such as tyrosine hydroxylase, acetylcholinesterase, DNA methyltrasferase, *etc.* [64,65]. Interestingly, rGTP concentrations in rabbit cells (estimations vary between 200 to 700 μM) [66,67] are slightly higher than the optimum concentration for maximum enzyme velocity, which supports the notion that these enzymes may operate under “substrate inhibition” conditions *in vivo*. A similar substrate inhibition phenomenon has previously been reported for other virus polymerases, including those of poliovirus [60], human immunodeficiency virus type 1 (HIV-1) [68] and hepatitis C virus (HCV) [69]. Notably, all these enzymes share a number of conserved motifs with rabbit calicivirus RdRps and seem to be structurally very similar [30]. Moreover, it was shown that, in case of the HCV RdRp, rGTP has a second low-affinity binding site outside the catalytic centre and it has been suggested that high rGTP concentrations suppress primer-dependent RNA synthesis and stimulate *de novo* RNA synthesis [69,70,71]. A similar mechanism, in which dNTPs act as non-competitive inhibitors, was proposed for the reverse transcriptase of HIV-1 [68]. For these enzymes, it is likely that substrate inhibition is a property that has evolved due to the fact that they have specialised sites where a second substrate molecule can bind and act as an allosteric regulator [65]. Considering the very conservative nature of viral RdRps [30], a similar regulatory role of rGTP may be proposed for rabbit calicivirus RdRps. It can be further speculated that a slower recovery of active enzymes due to substrate inhibition [64] may represent a speed-limiting mechanism to increase the fidelity of RNA synthesis thereby preventing a so-called “error catastrophe”, an outcome in which a genetic element cannot be maintained in a population as the fidelity of its replication machinery decreases beyond a certain threshold value [51,72,73,74]. The analysis of substrate inhibition, irrespective of whether it occurs under physiological or non-physiological conditions, can provide important insights into the functioning and regulation of enzymes [64].

### 4.2. Testing of RdRp Inhibitors

Eltahla *et al.* [45] and Yi *et al.* [46] demonstrated that *in vitro* fluorescence-based and gel-based polymerase activity assays can be used for the initial screening and testing of small compound inhibitors against positive-sense RNA viruses that cannot be grown in cell culture [44,45,46]. In this study, a total of seven potential rabbit calicivirus antivirals were tested.

NIC02 demonstrated inhibitory activity in the low micromolar range against rabbit calicivirus RdRps and can be considered as a scaffold for further development of therapeutic molecules against RHDV. NIC02 was previously reported as a viral NNI with no substantial effect on an RNA-dependent DNA polymerase (from avian myeloblastosis virus) and a DNA-dependent DNA polymerase (Taq polymerase). NIC02 demonstrated mixed mechanism of inhibition and activity against various species within the *Norovirus* and *Sapovirus* genera of the *Caliciviridae* family, and it has been suggested that such a broad inhibitory activity spectrum could be explained by putative binding of NIC02 to a highly conserved motif of calicivirus RdRps. NIC02 inhibitory effect on NoV replication was also validated in available cell culture systems (murine norovirus and GI.1 NoV replicon) [44].

In contrast to NIC02, the other six compounds that were tested against RCV and RHDV RdRps, namely NIC10, NIC12, NIC12-2, NIC12-3, NIC12-4 and NIC12-5 (all structural derivatives of NIC12) showed only modest or no inhibitory activities. These findings are in agreement with a previous study in which the inhibitory profiles of NIC10 and NIC12 were found to be restricted to particular RdRps from certain human NoV species [44].

## 5. Conclusions

The sudden emergence of RHDV2, a variant that could overcome protection afforded by existing vaccines, and the likely risk that even more virulent RHDV variants may emerge over time, highlight the need to develop alternative approaches to protect valued rabbit stocks and pets. The availability of effective antiviral agents against a broad range of RHDV variants would enable rabbit farmers to prevent/control RHD outbreaks more effectively. Using fluorescence-based and gel-based *in vitro* polymerase assays, we have identified NIC02 as a compound with inhibitory activity in the low micromolar range against rabbit calicivirus RdRps. NIC02 can be explored further as a potential scaffold in order to design a potent NNI capable of combating RHDV infections. Moreover, our results revealed a number of interesting properties of the RHDV and RCV RdRps that may help to better understand some of the unique characteristics of rabbit calicivirus pathobiology.

## Figures and Tables

**Table 1:** Sequences of primer pairs for generation of expression constructs.

Construct Name	Primer Sequence (5′→3′) ^a^
RdRp (RHDV)_His_6_	F: **ATG**ACGTCAAACTTCTTCTGTGG
	R: TATCCTCGAGCTCCATAACATTCACAAATTCGTC
RdRp (RCV)_His_6_	F: **ATG**ACTGCAAACTTCTTCTGTG
	R: TATCCTCGAGCTCCATAACATTCACAAAATCGTC
RdRp (NoV)_His_6_	F: TTTAAGAAGGAGATATACATATGGGAGGTGACAGTAAAGGGAC
	R: CAGTGGTGGTGGTGGTGGTGCTCGAGCTCGACGCCATCTTCATTC

^a^ RHDV Czech strain V351, RCV-A1 MIC-07 and NoV NSW028D/January/2013 genome sequences (GenBank accession numbers KF594473.1, EU871528.1 and KT239579, respectively) were used to design primers for PCR amplification. F—forward primer; R—reverse primer. *Nde*I and *Xho*I restriction sites are underlined. Initiation codon sequences are shown in bold.

**Table 2:** Kinetic analysis of the RHDV, RCV and NoV RdRps.

Biochemical Property	RHDV RdRp	RCV RdRp	NoV RdRp
*V*_max_ (pg(dsRNA) × min ^−1^ × ng(RdRp) ^−1^) at 30 °C	4.0 ± 0.4	13.4 ± 1.5	4.1 ± 0.6
*V*_max_ (pg(dsRNA) × min ^−1^ × ng(RdRp) ^−1^) at 42 °C (RHDV/RCV) or 37 °C (NoV)	6.6 ± 0.6	30.3 ± 1.3	5.8 ± 0.3
*K*_m_ (poly(C)) (ng/μL) at 30 °C	0.5 ± 0.1	0.9 ± 0.2	3.6 ± 0.6
*K*_m_ (rGTP), Michaelis-Menten model (μM) at 30 °C	14.6 ± 2.4	5.7 ± 0.7	77.7 ± 14.5
*K*_m_ (rGTP), substrate inhibition model (μM) at 30 °C	19.1 ± 3.8	8.0 ± 0.8	448.8 ± 120.4
*K*_i_ (rGTP), substrate inhibition model (mM) at 30 °C	2.1 ± 0.4	1.7 ± 0.2	0.6 ± 0.2
